# Effect of intraperitoneal cannabidiol (CBD) injection on intestine microbiome profile in a mouse model

**DOI:** 10.1007/s00294-025-01327-8

**Published:** 2025-09-30

**Authors:** Igor Jasielczuk, Ewa Ocłoń, Jakub Żurowski, Tomasz Szmatoła, Karolina Mizera-Szpilka, Artur Gurgul

**Affiliations:** 1 Department of Basic Sciences, Faculty of Veterinary Medicine, University of Agriculture in Krakow, Redzina 1c, 30-248 Krakow, Poland; 2 Laboratory of Recombinant Proteins Production, University of Agriculture in Krakow, Krakow, Poland; 3 Department of Infectious Diseases and Public Health, Faculty of Veterinary Medicine, University of Agriculture in Krakow, Mickiewicza 24/28, 30-059 Krakow, Poland

**Keywords:** Cannabidiol, Microbiome, 16S rRNA, Small intestine, Duodenum

## Abstract

Cannabidiol (CBD) as an active ingredient is widely used in cosmetics, food, medical products, and dietary supplements. Recent studies suggest that CBD consumption may influence the gut microbiome, impacting overall health. The mechanism of CBD action on the intestinal microbiome may be linked to the endocannabinoid system, which plays a role in the normalization of the functions of the immune system. Additionally, CBD may have a direct influence on the gut by interacting with the intestinal microflora, regulating intestinal permeability, modulating immune responses, and affecting the gut-brain axis communication through the vagal signaling pathway. A study was conducted to analyze how different doses of CBD injected intraperitoneal could alter the microbial composition of the duodenum contents in a mouse model. Obtained results suggest that the impact of CBD on the intestine microbiome may be dosage-dependent. Statistical analysis revealed significant differences compared to the control group in the 0.2 mg/kg CBD and 20 mg/kg CBD dose groups for the genera Campilobacteriota, Firmicutes, Proteobacteria, and, specifically in the 0.2 mg CBD group, Cyanobacteria. In contrast, the 10 mg CBD dose group showed no statistically significant differences relative to the control. The Firmicutes phylum had the highest relative abundance in all groups, wherein its share was lower in the CBD-treated groups than in the control group. *Lachnospiraceae*_*NK4A136*_*group*, *Muribaculaceae*, and *Akkermansia* were predominant, with *Lachnospiraceae_NK4A136_group* decreasing and *Akkermansia* increasing in relative abundance in CBD-treated groups. Of the genera that showed statistically significant differences between groups, most belonged to the phylum Firmicutes, including the dominant *Lachnospiraceae_NK4A136_group*. These findings suggest that intraperitoneal CBD administration induces alterations in the duodenal microbiome, particularly affecting the *Lachnospiraceae_NK4A136_group* and the *Akkermansia* genera.

**Supplementary Information:**

The online version contains supplementary material available at 10.1007/s00294-025-01327-8.

## Background

Cannabidiol (CBD) is a phytocannabinoid obtained from the hemp species (mainly *Cannabis sativa*), which does not have psychomimetic properties (Morris et al. 2020). Numerous scientific studies indicate that its effect on the human body may be analgesic, anticonvulsant, muscle relaxant, anxiolytic and antipsychotic. CBD has also been confirmed to have neuroprotective, anti-inflammatory, and antioxidant effects (Peng et al. 2022). Recent reports suggest that CBD consumption may affect the quantitative and qualitative composition of the gastrointestinal microflora and thus affect the health and condition of the body (Ibrahim et al. 2022). CBD as an active agent is used in the production of cosmetic, food and medical products and dietary supplements (Cerino et al. 2021). Commercially available CBD products used by humans come in many forms (e.g. oils, solutions, capsules, sublingual sprays, e-cigarette liquids) and are characterized by various forms of administration: oral, inhalation, or topical (Javadi-Paydar et al. 2019; Souza et al. 2022). What is more, CBD as an additive to pet food and treats is now widely available and increasingly used in companion animals (Bradley et al. 2022). Due to the ever-growing interest in CBD supplementation both in humans and animals, there is an urgent need for research to evaluate both its safety and effectiveness especially regarding the effect on the liver, gastrointestinal tract, endocrine system, nervous system, psychological functions, and reproductive system (Lachenmeier et al. 2023; Henderson et al. 2023; Morris et al. 2020). It is worth noting that despite the presence of CBD-containing products on the market, there has been little research on the safety of long-term CBD intake, and the likelihood of such consumer exposure is high (Skinner et al. 2020).

The pathway of CBD effects on the gut microbiome can be related to endogenous endocannabinoid system (Karoly et al. 2020), which is involved in the normalization of the functions of the immune system (Nagarkatti et al. 2009). What is more, CBD can directly affect the intestines (Izzo and Sharkey 2010); Trautmann and Sharkey 2015) by interacting with the intestinal microflora (Cani et al. 2016), regulating intestinal permeability (Muccioli et al. 2010), modulation of immune responses (Nagarkatti et al. 2009), and effects on vagal signaling on the gut-brain axis (Storr and Sharkey 2007). In the available literature, no research results show the direct impact of different concentrations of CBD alone (chemically pure) on the composition of the intestinal microbiome (Oliveira et al. 2022). The available study results concern the effect of CBD on bacterial microbiome changes in mice with induced colitis (Silvestri et al. 2020). In this study, however, the authors used a relatively low dose of CBD (1 mg/kg), focusing mainly on the effect of CBD on intestinal inflammation. Despite this, they showed that even such a low CBD dose caused changes in the microbiome profile (Silvestri et al. 2020). Gorelick et al. (2022) determined the effect of CBD (2.39 mg/kg) on the composition of the intestinal microflora of mice subjected to a high-fat and cholesterol diet (HFCD). Al-Ghezi et al. (2019) elucidated alterations in the intestine microbiome resulting from the administration of a combination of THC and CBD in a 1:1 ratio during experimental autoimmune encephalomyelitis. Simultaneously, Skinner et al. (2020) scrutinized the impacts of a cannabidiol-rich cannabis extract (CRCE) on the murine gut microbiome. Their findings revealed intricate responses induced by CRCE, with potential outcomes ranging from beneficial to harmful. Notably, CRCE was observed to enhance the abundance of *A. muciniphila*, a bacterial strain known for its probiotic properties. This observation raises concerns about the potential long-term effects of CBD ingestion. Additionally, there are several studies available on the use of CBD in gastrointestinal disorders, however, none of these studies analyzed changes in the composition of the intestinal microflora (Couch et al. 2018; Couch et al. 2019; Martínez et al. 2020; Naftali et al. 2017; van Orten-Luiten et al. 2022).

To the best of our knowledge, the available literature lacks the results of studies on the effect of CBD on the microbiome in healthy individuals consuming CBD by systemic (not oral route) in various doses. A popular form of non-oral delivering CBD to the body is smoking hemp flowers or using e-cigarette liquids. To our current understanding, the literature available does not present data regarding the potential effects of this mode of consumption on the intestinal microflora. Javadi-Paydar et al. (2019) demonstrated that inhaling CBD results in similar blood plasma levels to those achieved through intraperitoneal injection of 10–30 mg/kg. We assume that CBD, when administered systemically—either by intraperitoneal injection or by inhalation —can affect various organs, including the immune system, which in turn may influence intestinal microbiota composition and function. The study aimed to conduct a comprehensive analysis of the impact of various doses of CBD, administered through intraperitoneal (IP) injection, on the qualitative and quantitative characteristics of the duodenum microbiome in a murine model. Duodenal content was selected for microbiome analysis due to: (Al-Ghezi et al. 2019) the high density of endocannabinoid receptors within its wall, making it a primary target for intraperitoneally administered CBD and a direct site for the potential modulation of the gut-brain axis (Pertwee and Ross 2002; Izzo and Sharkey 2010; Alhouayek and Muccioli 2012) its pivotal role in modulating motility and digestive secretion, which indirectly shape the microbial community (Alhouayek and Muccioli 2012; Al Shoyaib et al. 2020) a unique and dynamic microbial profile characterized by lower biomass but high sensitivity to host homeostasis disruptions, thereby enabling the detection of subtle, dose-dependent effects of eCB modulation (Zoetendal et al. 2012; Booijink et al. 2010); and (Bolyen et al. 2019) the feasibility of precise and reproducible sample collection in the animal model, which minimizes variability arising from environmental heterogeneity in the distal segments of the gastrointestinal tract.

## Materials and methods

### Study population and settings

Approximately 60 day old male C57BL/6J mice (weighing 20–25 g) were obtained from the animal facility of the Department of Clinical Immunology and Transplantology, Institute of Pediatrics, Jagiellonian University Medical College and housed per experimental group (6 animals randomly allocated to each group) in polycarbonate cages (40 × 25 × 22 cm), under a 12-h light/dark cycle, controlled temperature (21 ± 2 °C) and constant humidity (60 ± 10%). Mice had free access to tap water and standard rodent feed (ZL-H specialist feed; Zoolab, Poland) (the composition of the feed is given in supplementary file 1). All animal procedures were approved by the Local Animal Care Ethics Committee No. II in Kraków–permission number 90/2022 in accordance with EU regulations.

The mice were randomly assigned to four groups (6 mice each) received different doses of CBD (group I – 0.2 mg/kg b.w.; group II – 10 mg/kg b.w.; group III – 20 mg/kg b.w.) and a placebo (vehicle; control group). The CBD injection period lasted 28 days. The posology was based on an overview of human clinical trials (Millar et al. 2019) and an overview of the recommendations available with commercially sold CBD supplements. The group receiving a dose of 0.2 mg/kg was included in the study to determine whether such a low systemic exposure would induce any changes in the duodenal microbiome. Chemically pure CBD was administered in a solution with 2% TWEEN 80 in saline (or 2% TWEEN 80 in saline as a placebo) as IP injection, in a single daily dose. Animals were fasted for 16 h after which anesthetized with isoflurane and immediately euthanized by decapitation.

## Samples preparation, nucleic acid isolation and 16 S rRNA sequencing

After euthanasia, DNA was isolated directly from the content of the duodenum using the QIAmp PowerFecal DNA kit (Qiagen, Hilden, Germany) according to the manufacturer’s instructions. Amplification of the V3 and V4 regions of the 16 S rRNA gene for bacteria was performed in accordance with the protocol for performing the 16 S Metagenomic Sequencing (Illumina, San Diego, CA, USA) libraries. The pooled library, with a 15% PhiX control DNA spike-in, was applied to the cartridge for sequencing. Sequencing was performed using the Reagent Kit V3 (600 cycles) in the MiSeq platform (Illumina) in a 2 × 300 bp paired-end run at the Faculty of Food Technology, University of Agriculture in Krakow, Poland. The obtained raw sequences were deposited in the Sequence Read Archive (SRA) database of the National Center for Biotechnology Information (NCBI) under accession number PRJNA1155312.

## Bioinformatics analysis and statistical analysis

The obtained raw sequencing reads were analyzed using QIIME2 (Bolyen et al. 2019) software in which initial reads quality control, filtering, denoising and feature table generation with the use of DADA2 (Callahan et al. 2016) software were performed successively. With the use of mafft (Katoh and Toh 2010) software and FastTree software (Price et al. 2009) on the masked alignment a phylogenetic tree has been generated. Based on refraction curve analysis, 3,000 sampled reads were selected for alpha and beta diversity analysis using the q2-diversity plugin. Alpha and beta diversity were assessed in MicrobiomeAnalyst software (Dhariwal et al. 2017). The alpha diversity was determined using the observed number of features, Shannon’s diversity index and Simpson index. Beta diversity was determined using Bray-Curtis distance, Jaccard distance and Jensen-Shannon divergence. Then, the alpha and beta diversity parameters were compared between groups using the Kruskal-Wallis or PERMANOVA tests, for alpha and beta diversity, respectively. Reads taxonomic classification was performed with a pre-trained Naive Bayes classifier (sklern) and the q2-feature-classifier plugin. The Weighted Silva-138 99% OTUs classifier was used as the reference for taxonomic assignments based on SILVA v138 database (Quast et al. 2012). With the use of MicrobiomeAnalyst (Dhariwal et al. 2017) software and the implemented EdgeR (Robinson et al. 2010) tool, differential abundance analysis among groups was carried out with a false discovery rate (FDR) < 0.05 for differentially abundant taxa.

## Results

### Sequencing statistics, biodiversity analysis, and taxonomic classification

Sequencing of 48 samples gave 2,333,646 reads, 97,235.3 (SD = 15,430) reads on average, per sample. The minimum number of reads per sample was 64,952 and the maximum was 126,354. After filtering, 1,041,816 reads remained, 43,409 (SD = 7,499) on average (from 26,727 to 57,074 reads per sample). The microbiota composition at the species level (L7) was not analyzed, due to the high possibility of error in the taxonomic assignment using 16 S V3-V4 regions analysis (Klindworth et al. 2013) and in the interpretation of the obtained data (the percentage of classified reads was below 80%) (Table 1).

**Table 1:** A summary of phylogenic classification

Taxonomic Level	No. of Classified Reads	Percent of Reads
Kingdom	1,041,816	100%
Phylum	1,041,797	100%
Class	1,041,797	100%
Order	1,041,770	100%
Family	1,041,759	99.99%
Genus	883,071	84.82%
Species	793,517	76.25%

**Fig. 1:**
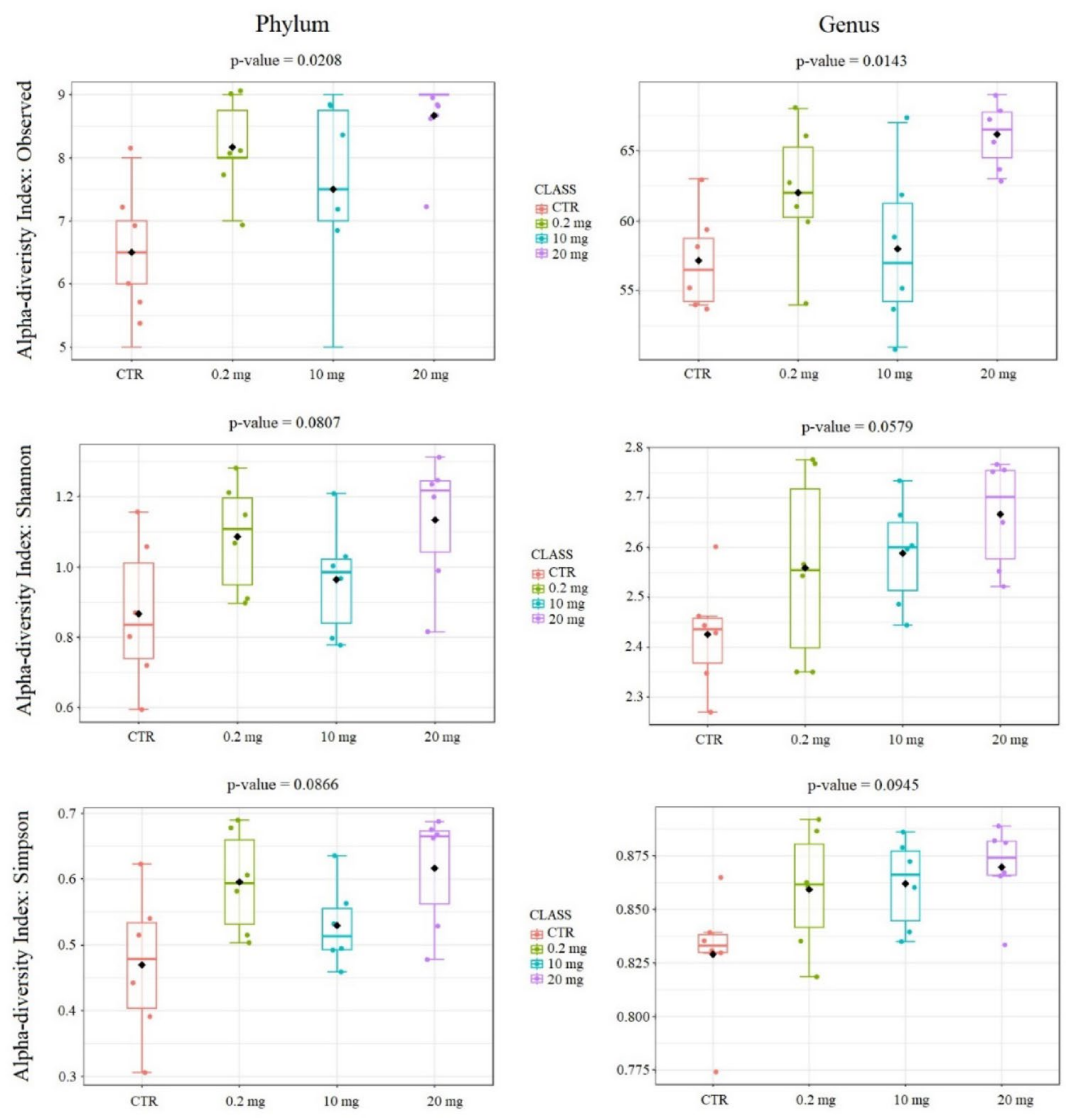
Alpha diversity indexed in the studied groups at the phylum and genus levels

On average, from 79 (10 mg supplementation) to 95 (20 mg supplementation) ASVs (amplicon sequence variants) were detected within the analyzed groups. Analysis of alpha diversity indexes, such as Shannon’s diversity index, Observed Features, and Simpson index showed that there were no statistically significant (q-value < 0.05) differences between analyzed groups in these parameters at the phylum level and at the genus level. Only observed features showed statistically significant differences in one specific comparison, namely between the control group (CTR) and 20 mg group. Figure 1 presents box plots for the alpha diversity indices used at the phylum and genus levels.

**Fig. 2:**
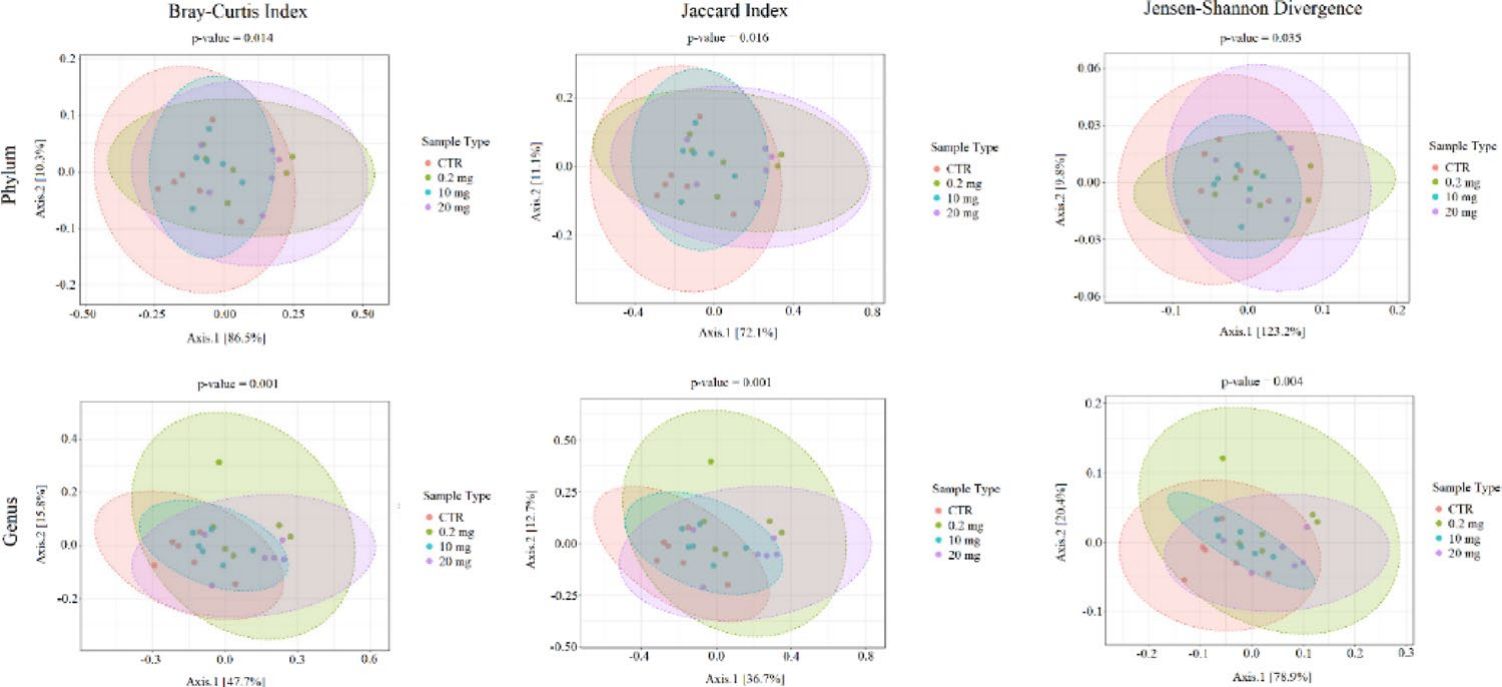
Beta diversity indexes in the studied groups at the phylum and genus levels

In case of beta diversity, at the phylum level the Bray-Curtis distance, the Jaccard distance and the Jensen Shannon Divergence did not show any statistically significant differences between all group pairs. At the genus level, the Bray-Cutris distance showed statistically significant differences between all pairs of groups except of 0.2 mg versus 10 mg and 0.2 mg versus 20 mg. The Jaccard distance showed statistically significant differences between the control group and all three CBD-treated groups. The Jensen Shannon Divergence showed statistically significant differences only between CTR versus 0.2 mg and CTR versus 20 mg. Figure 2 shows plots for the beta diversity indices used at the phylum and genus levels. Table 2 presents q-values for all comparisons between the analyzed groups for the alpha and the beta diversity parameters used.

**Table 2:** Alpha and beta diversity parameters q-values for comparisons between the analyzed groups (q < 0.05 is considered significant)

Taxonomic level	Diversity	Index	CTR vs. 0.2 mg (q-value)	CTR vs. 10 mg (q-value)	CTR vs. 20 mg (q-value)	0.2 mg vs. 10 mg (q-value)	0.2 mg vs. 20 mg (q-value)	10 mg vs. 20 mg (q-value)
Phylum	Alpha diversity	Observed	0.063	0.262	0.056	0.503	0.262	0.251
		Shannon	0.195	0.589	0.195	0.360	0.589	0.264
		Simpson	0.198	0.473	0.198	0.198	0.937	0.198
	Beta diversity	Bray-Curtis	0.096	0.203	0.096	0.203	0.833	0.096
		Jaccard	0.102	0.216	0.102	0.216	0.8	0.102
		Jensen-Shannon	0.165	0.472	0.138	0.263	0.764	0.178
Genus	Alpha diversity	Observed	0.161	0.936	0.038	0.314	0.161	0.074
		Shannon	0.360	0.078	0.052	0.818	0.818	0.360
		Simpson	0.481	0.123	0.091	0.937	0.706	0.706
	Beta diversity	Bray-Curtis	0.03	0.036	0.033	0.059	0.314	0.042
		Jaccard	0.033	0.036	0.033	0.059	0.374	0.051
		Jensen-Shannon	0.036	0.096	0.036	0.109	0.296	0.096

### Relative abundance

**Fig. 3:**
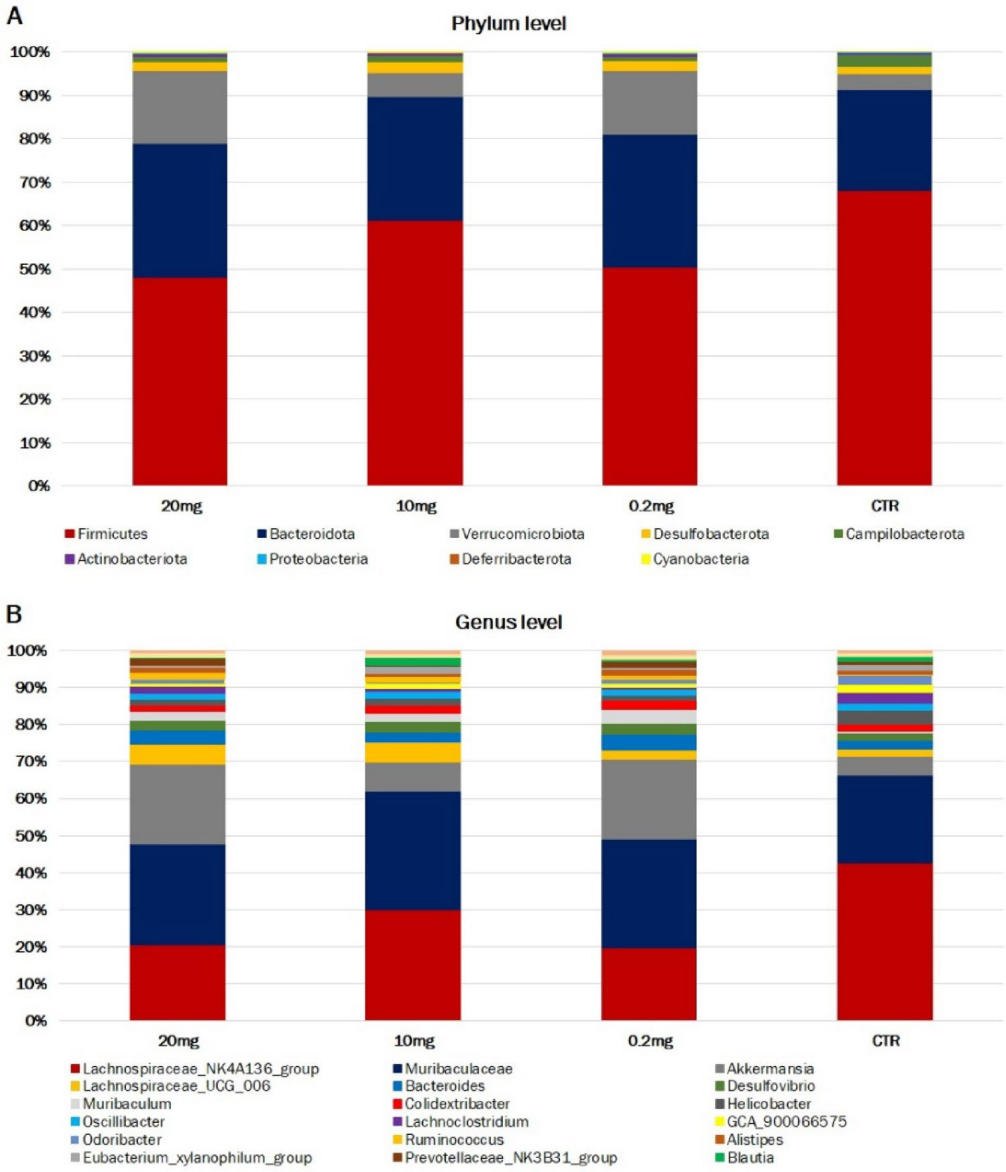
(A) Relative abundance of 20 most represented bacterial phyla. (B) Relative abundance of 20 most represented bacterial genera

The assessment of the murine intestine microbiome was carried out at the taxonomic level of phylum (L2) to species (L7), two of which are described in this report: L2 (phylum) and L6 (genus). At the phylum level (L2), all ASVs obtained for the analyzed groups were assigned to nine phyla: Firmicutes, Bacteroidota, Verrucomicrobiota, Desulfobacterota, Campilobacterota, Actinobacteriota, Proteobacteria, Cyanobacteria and Deferribacterota. The phylum Firmicutes was predominant in each analyzed group (from 48.2% in 20 mg CBD to 68.2% in CTR). The share of Bacteroidota (from 23.2% in CTR to 30.9% in 20 mg CBD) and Verrucomicrobiota (from 3.6% in CTR to 16.6% in 20 mg CBD) was large, while other phyla accounted for negligible amounts, as shown in Fig. 3A. At the genus level, all ASVs identified were assigned to 66 genera. For all the analyzed groups, only three genera were characterized by a share of more than 10% in at least one group: *Lachnospiraceae_NK4A136_group* (from 13.6% in 0.2 mg to 31.1% in CTR), *Muribaculaceae* (from 17.4% in CTR to 23.3% in 10 mg CBD) and *Akkermansia* (from 3.6% in CTR to 16.6% in 20 mg CBD) (Fig. 3B). The percentage taxonomic composition of the analyzed groups at the Phylum and Genus levels is presented in Online Supplementary File 1, Table S1 and Table S2, respectively.

## Differential analysis

Analyzing pairwise comparisons of the analyzed groups, the highest number of statistically significant differences at the phylum level have been found among 0.2 mg CBD and CTR groups whereby an increase in abundance has been shown for Proteobacteria (log2FC = 2.7, FDR = 0.0199) and Cyanobacteria (log2FC = 2.6, FDR = 0.0199). The decrease was observed for Campilobacterota (log2FC=−2.6, FDR = 0.0199) and Firmicutes (log2FC=−1.1; FDR = 0.0199) phyla share in 0.2 mg CBD treated group. Furthermore, statistically significant increase of Proteobacteria (log2FC = 2.3, FDR = 0.0407) and decrease of Campilobacterota (log2FC=−2.6, FDR = 0.0114) and Firmicutes (log2FC=−1.4, FDR = 0.0046) at the phylum level also have been found in comparison between 20 mg CBD vs. CTR groups. Consecutively, Proteobacteria showed statistically significant decrease in 10 mg CBD-treated group compared to 0.2 mg CBD-treated group (log2FC=−2.8, FDR = 0.0327).

The highest number of statistically significant different genera have been found among 0.2 mg CBD and CTR (13 genera) and 20 mg CBD and CTR (12 genera). The highest number of highly statistically significant differences between pairs of the analyzed groups were found for *Staphylococcus* genus for which a statistically significant decrease was noted compared to the control group for doses of 0.2 mg CBD (log2FC=−6.5, FDR = 3.9E-7), 10 mg CBD (log2FC=−2.8, FDR = 0.0442) and 20 mg CBD (log2FC=−3.3, FDR = 0.0129). Moreover, compared to the 0.2 mg CBD dose, a statistically significantly greater share of this genus was observed for the 10 mg CBD (log2FC = 3.7, FDR = 0.0042) and 20 mg CBD doses (log2FC = 3.3, FDR = 0.0235). *Dubosiella* genus showed highly statistically significant increase relative to CTR for 0.2 mg CBD dose (log2FC = 7.8, FDR = 2.19E-05) and the 20 mg CBD dose (log2FC = 9.1, FDR = 3.93E-06). In addition, there was a statistically significant increase of this genus for the 20 mg CBD group compared to the 10 mg CBD group (log2FC = 9.0, FDR = 4.28E-06) and a statistically significant decrease for the 10 mg CBD dose compared to the 0.2 mg CBD group (log2FC=−7.7, FDR = 0.0001). Apart from the *Staphylococcus* genus, only two genera showed a statistically significant change for all three CBD-treated groups relative to the CTR group. The *Tuzzerella* genus showed a statistically significant increase, and the *Jeotgalicoccus* genus showed a statistically significant decrease relative to the control group. Statistically significant differences in abundance at the phylum and the genus levels between individual pairs of treated groups are presented in Online Supplementary File 1, Table S3.

## Discussion

Nowadays, a growing amount of scientific evidence indicates that phytocannabinoids may influence healthy gut flora, communication between the gut and the brain, and overall strengthen gut health (Cani et al. 2016; Karoly et al. 2020). Despite the dynamic increase in the popularity and consumption of products containing CBD, there are few research results available related to the impact of CBD on the intestinal microbiome. To the best of our knowledge, there are no studies in the available literature determining the effect of CBD on the microbiome of the small intestine, which is characterized by a dynamic environment that is less diverse and less populated by microorganisms compared to the colonic microflora (Sender et al. 2016; Martinez-Guryn et al. 2019; Yersin et al. 2024). Nevertheless, over the last few years, a growing interest in small intestinal bacterial overgrowth (SIBO) disorder studies is observed, making the small intestine an important subject of research (Efremova et al., 2023). Our study aimed to determine the effect of different doses (0.2–20 mg/kg) of CBD administered by intraperitoneal injection (simulating other than the oral route of CBD administration) on the bacterial microbiome of the duodenum. We used the injection form of CBD administration because it has a systemic effect on the body, similar to inhalation, which is one of the most popular ways of delivering CBD to the body and allows for precise dose measurement (Shoyaib et al. 2020).

This study showed statistically significant differences between the analyzed groups at both the phylum and genus levels. Among CBD-treated groups, 0.2 mg CBD group showed the largest number of statistically significantly changed phyla compared to the control group. The Firmicutes phylum had the highest relative abundance for each group, however its share in the CBD-treated groups was lower than in the control group and this is associated with an increase in the share of the Verrucomicrobiota, Bacteroidota and Desulfobacterota phyla. What is more, the groups receiving a dose of 0.2 mg CBD and 20 mg CBD showed a statistically significant decrease in the share of the Firmucutes phylum compared to the control group. Apart from the Firmicutes phylum, statistically significant changes concerned phyla characterized by a negligible share. It is worth noticing, that throughout our study, the relative abundance profile of microbiota in the 10 mg CBD dose-treated group differed from the profiles of the other CBD-treated groups and was more similar to that of the control group. At the phylum level, no significant differences were found between these groups. This suggests that CBD effect on gut microbiome might be dosage-dependent (as previously presented for other analyzed physiological traits (Millar et al. 2019) or some unidentified factors appeared in 10 mg CBD-treated group that affected their gut microbiome.

The taxonomic composition of the studied groups at the genus level was dominated by *Lachnospiraceae_NK4A136_group*, *Muribaculaceae* and *Akkermansia*, which accounted for about 50% of the relative abundance. Several genera exhibited statistically significant differences between the analyzed groups, most of which belonged to the phylum Firmicutes. The decrease in the relative abundance of Firmicutes in the CBD-treated groups compared to the control group was largely attributed to a reduction in the *Lachnospiraceae_NK4A136_group*. However, a statistically significant decrease for this genus was observed only between the group treated with 0.2 mg CBD and the control group. The *Lachnospiraceae_NK4A136_group* is a genus of bacteria belonging to the family *Lachnospiraceae*. These bacteria are Gram-positive, and anaerobic, and their primary role in the gut is the fermentation of dietary fibres, which leads to the production of short-chain fatty acids (SCFAs), such as butyrate, acetate, and propionate. (Fusco et al. 2023; Li et al.,^2022^ Wu et al., 2020). The *Lachnospiraceae_NK4A136_group* is known to produce substantial amounts of butyrate, which plays a key role in regulating gut inflammation and the maturation of the immune system (Ćesić et al. 2023; Kasahara et al. 2018). An increased abundance of the *Lachnospiraceae_NK4A136_group* has been observed in mouse models of colitis and diet-induced obesity, indicating a potential involvement in disease-related inflammatory processes (Kim et al., 2021; Liu et al. 2019; Shao et al. 2020). Similarly, the endocannabinoid (eCB) system in the gut has been linked to the regulation of gut inflammation and metabolism. A cannabinoid receptor 1 (CB1) antagonists modulate intestine microbiome composition, decreases inflammatory responses and pro-inflammatory macrophage presence in adipose tissue, and lower plasma LPS levels, consequently reducing both intestinal permeability and metabolic endotoxemia (Mehrpouya-Bahrami et al. 2017). The decrease in the relative abundance of *Lachnospiraceae_NK4A136_group* may be associated with CBD’s action as a negative allosteric modulator of the CB1 cannabinoid receptor, reducing the ability of other substances to activate this receptor and thereby attenuating the CB1 response to compounds that would typically stimulate it strongly. Through its effects on CB1, CBD may limit certain metabolic outcomes associated with excessive CB1 activation, such as increased appetite, fat storage, and inflammation in peripheral tissues, including the intestines. Mehrpouya-Bahrami et al. (2017) investigated the effect of treating mice with a cannabinoid receptor 1 (CB1) antagonist (SR141716A, Rimonabant) on diet-induced obesity and demonstrated that CB1 blockade drastically increased the relative abundance of *Akkermansia muciniphila* while reducing *Lachnospiraceae* and *Erysipelotrichaceae* in the gut confirming the eCB system-mediated changes in microbiota composition. The authors suggested that CB1 blockade alleviates diet-induced obesity and metabolic disorders by modulating macrophage inflammatory mediators, and this effect is linked to changes in the gut microbiome and its metabolites (Mehrpouya-Bahrami et al. 2017). Wang et al. (2024) demonstrated in a mouse model of obstructive sleep apnea that CB1 receptor inhibition increased the diversity of colonic bacterial flora and regulated intestine microbiome composition. Additionally, the concentration of butyric acid and the abundance of SCFA-producing bacteria, such as *Ruminococcaceae* and *Lachnospiraceae*, were significantly elevated by CB1 receptor inhibition. The authors suggested that CB1 receptor inhibition could mitigate colon damage caused by chronic intermittent hypoxia by modulating the gut microbiota, reducing mucosal injury, and promoting tight junction regeneration (Wang et al. 2024). It is noteworthy that, in our study, alongside the observed increase in the relative abundance of genera such as *Lachnospiraceae_NK4A136_group*, *Lachnoclostridium*, and *GCA_900066575*, a decrease in the genus *Lachnospiraceae_UCG_006* was also detected. This finding may indicate a dual effect of CBD on genera within the *Lachnospiraceae* family.

*The Akkermansia* genus was characterized by an increase in relative abundance in CBD-treated groups relative to the control, especially in the groups treated with the lowest and highest doses of CBD. The genus *Akkermansia* belongs to the phylum Verrucomicrobia, and its only known representative occurring in feces is *Akkermansia muciniphila* (Silvestri et al. 2020; Markowska and Kiersztan 2021). A. muciniphila has a direct and indirect impact on the host organism, marked by its characteristic ability to degrade on of the components of the intestinal mucosa- mucin. (Markowska and Kiersztan 2021). Through the constant breakdown of mucin, it significantly stimulates its new production, enabling the maintenance of the appropriate thickness of the mucous membrane, which has a positive effect on the functioning of the intestinal barrier, preventing the translocation of pathogenic bacteria deep into the tissues. By interacting with other bacteria, *A. muciniphila* contributes to their production of many beneficial metabolites (including SCFAs) and prevents excessive concentration of harmful ones (e.g. hydrogen sulfide) (Markowska and Kiersztan 2021). The increase in the share of *Akkermansia* observed in our study in the CBD-treated groups compared to the control group is similar to the results of the majority of previous studies. However, Silvestri et al. (2020) showed that in the DSS mouse model of colitis, regardless of the inflammation state, under the influence of both CBD (1 mg/kg) and fish oil (20 mg/kg) administered together and separately, there was an increase in the share of *Akkermansia muciniphila*. Skinner et al. (2020) showed an increase in the share of *Akkermansia muciniphila* under the influence of high doses of CRCE. In turn, Gorelick et al. (2022) in their study showed no changes for the *Akkermansia* genus as a result of CBD administration. On the contrary, Al-Ghezi et al. (2019) showed that the combination of THC and CBD (intraperitoneal injection of 10 mg/kg each of THC + CBD) significantly reduced the abundance of *Akkermansia muciniphila* in the gut in a mouse model of experimental autoimmune encephalomyelitis. Both our and the previous studies indicate an influence of CBD (alone or with additives) on the abundance of the genus *Akkermansia* in the intestines, but in an ambiguous way. This may be due to many factors differentiating the discussed study results. Material for analysis in the current and mentioned studies was collected in different anatomic regions of the intestinal tract. Physiological changes occurring in various areas of the small intestine and colon (chemical and nutritional gradients and isolated host immune activity) are thought to influence the composition of bacterial communities (Donaldson et al. 2016, Tang et al. 2020). In our study, we collected the contents of the duodenum. In turn, Al-Ghezi et al. (2019) and Gorelick et al. (2022) collected the contents of the cecum, and Silvestri et al. (2020) and Skinner et al. (2020) collected the contents of the colon. Furthermore, both in our study and in the study of Al-Ghezi et al. (2019), CBD was administered by intraperitoneal injection, and Silvestri et al. (2020), Skinner et al. (2020), and Gorelick et al. (2022) used oral administration. What’s more, the potential therapeutic effects of individual phytocannabinoids are different from the effects of the mixtures containing various compounds derived from hemp (Gorelick et al. 2022). Al-Ghezi et al. (2019) administered a combination of CBD and THC, while Skinner et al. (2020) did not administer chemically pure CBD, only CRCE, and in huge doses (61.5–615 mg/kg) compared to those used in our studies. In turn, Silvestri et al. (2020) conducted their study in a DSS model of murine colitis, which also makes direct comparison difficult.

An interesting observation from this study is also the effect of CBD on the genus *Helicobacter*. All doses of CBD led to a decrease in the relative abundance of this genus compared to the control group; however, this reduction was not statistically significant. Furthermore, it should be noted that a significant percentage of reads (ranging from 10% in the 20 mg CBD group to 19% in the 0.2 mg CBD group) were not assigned to any taxon, resulting in incomplete data on microbiome profile shifts in the mouse duodenum. As new taxa are identified and 16 S rRNA databases expand, it will become possible to supplement our current, partial understanding. Additionally, future studies examining microbiome changes across various intestinal sections could provide a more comprehensive profile of CBD’s effects on the gut. It would also be advantageous to analyze the entire 16 S unit rather than just the v3 and v4 regions, allowing for taxonomic identification at the species level, unlike in our study, where taxa were only classified to the genus level.

## Conclusions

This study describes the impact of various dosages of CBD on the bacterial microbiome in the duodenum. The obtained results suggest that CBD effect on the intestinal microbiome might be dosage-dependent. The group receiving the lowest dose of CBD (0.2 mg/kg) had a higher number of phyla that experienced statistically significant changes compared to the control group. The Firmicutes phylum was characterized by the highest relative abundance for each group, wherein its share in the CBD-treated groups was lower than in the control group. At the genus level, the taxonomic composition of the examined groups was primarily dominated by *Lachnospiraceae_NK4A136_group*, Muribaculaceae, and *Akkermansia*, together accounting for nearly half of the overall relative abundance. Several genera displayed statistically significant variations among the groups, with most belonging to the Firmicutes phylum. The observed decrease in Firmicutes relative abundance within the CBD-treated groups, compared to controls, was largely due to a decline in the *Lachnospiraceae_NK4A136_group* genus. *Akkermansia* exhibited an increase in relative abundance in the CBD-treated groups in compared to the control group.

## Supplementary Information

Below is the link to the electronic supplementary material.

Supplementary Material 1

## Data Availability

The obtained raw sequences were deposited in the Sequence Read Archive (SRA) database of the National Center for Biotechnology Information (NCBI) under accession number PRJNA1155312.
